# Clinical and aetiological study of hand, foot and mouth disease in southern Vietnam, 2013–2015: Inpatients and outpatients

**DOI:** 10.1016/j.ijid.2018.12.004

**Published:** 2019-03

**Authors:** Minh Tu Van Hoang, To Anh Nguyen, Tan Thanh Tran, Thi Ty Hang Vu, Nguyen Truc Nhu Le, Thi Han Ny Nguyen, Thanh Hoang Nhat Le, Thi Thu Hong Nguyen, Thanh Hung Nguyen, Nguyen Thanh Nhan Le, Huu Khanh Truong, Tuan Quy Du, Manh Tuan Ha, Lu Viet Ho, Chau Viet Do, Tran Nam Nguyen, Thi My Thanh Nguyen, Saraswathy Sabanathan, Tu Qui Phan, Vinh Chau Nguyen Van, Guy E. Thwaites, Bridget Wills, C. Louise Thwaites, Van Tan Le, H. Rogier van Doorn

**Affiliations:** a Oxford University Clinical Research Unit, Ho Chi Minh City, Vietnam; b Children’s Hospital 2, Ho Chi Minh City, Vietnam; c Children’s Hospital 1, Ho Chi Minh City, Vietnam; d Hospital of Tropical Diseases, Ho Chi Minh City, Vietnam; e Centre for Tropical Medicine and Global Health, Nuffield Department of Medicine, University of Oxford, Oxford, UK

**Keywords:** Hand, Foot and mouth disease, Enterovirus, Vietnam, Enterovirus A71, Coxsackievirus

## Abstract

**Background:**

Hand, foot and mouth disease (HFMD) has been associated with large outbreaks among young children in the Asia-Pacific Region since 1997, including cases of severe illness and death. Severe illness is often associated with enterovirus A71 (EV-A71). Vietnam experienced a large sustained outbreak of 200 000 hospitalized cases and over 200 deaths in 2011–12, the large majority occurring in southern Vietnam.

**Methods:**

A prospective observational study was conducted in the outpatient clinics, infectious diseases wards, and paediatric intensive care units of the three main referral centres for the treatment of HFMD in southern Vietnam. Demographic data, basic laboratory parameters, and clinical data were recorded, and molecular diagnostic tests were performed.

**Results:**

Between July 2013 and July 2015, a total of 1547 children were enrolled. Four serotypes of enterovirus A (EV-A71, Coxsackievirus (CV) A6, A10, and A16) were responsible for 1005 of 1327 diagnosed cases (75.7%). An unexpected dominance of EV-A71 was found among both inpatients and outpatients, as well as a strong association with severe illness. CV-A6 and CV-A10 emerged in Vietnam during the study period and replaced CV-A16. CV-A10 was associated with different clinical and laboratory characteristics. During admission, 119 children developed a more severe illness. It was found that children with a skin rash showed less progression of severity, but when a rash was present, a macular rash was significantly associated with an increased risk of progression.

**Conclusions:**

This study represents the most comprehensive descriptive HFMD study from Vietnam to date. Co-circulation and replacement of different serotypes has implications for vaccine development and implementation. These findings from a severely affected country add to our understanding of the presentation, progression, and aetiology of HFMD.

## Highlights

• Multiple serotypes of enterovirus A cause hand, foot and mouth disease in southern Vietnam.
• Clinical characteristics differed slightly between the different pathogen groups.
• CV-A6 and CV-A10 emerged in Vietnam in 2013–2015.
• An unexpected dominance of EV-A71 was found among both inpatients and outpatients.

## Introduction

Hand, foot and mouth disease (HFMD) is a common, mild viral infection that mostly affects children under 5 years of age. The causative agent is typically enterovirus A (EV-A), which includes the serotypes Coxsackievirus A (CV-A) 2–8, 10, 12, 14, and 16 and enterovirus A71, A76, and A89–92; serotypes of enterovirus B are sometimes detected (Wang et al., 2018, Yang et al., 2011, Park et al., 2011). Since 1997 attention has focused on the Asia-Pacific region due to the occurrence of several large outbreaks, with millions of reported cases, including a small proportion of cases progressing to severe disease and even death (Chan et al., 2000, Ho et al., 1999, Zhang et al., 2010, Khanh et al., 2012). Many countries in the Asia-Pacific region now have established surveillance systems for HFMD, including China, Singapore, Taiwan, Malaysia, and Vietnam. The incidence of EV-A71-associated HFMD has increased dramatically since the Sarawak outbreak in 1997, although it varies according to country and year (Zhao et al., 2015).

Currently, there is no specific antiviral treatment for HFMD, but as the causative agent of the large outbreaks with severe illness has been EV-A71, attention has focused particularly on agents active against this virus. Several antivirals with activity against enteroviruses (including EV-A71) have been developed, but few have undergone extensive clinical evaluation (Tian et al., 2012). Vaccine development has been more successful with the completion of three phase 3 trials in 2013–14 and registration of two inactivated EV-A71 vaccines in China in 2016 (Zhu et al., 2013, Zhu et al., 2014). These vaccines have an efficacy of over 90% against EV-A71-associated HFMD, but weak protection against HFMD caused by other circulating EV-A serotypes. Protection against severe disease and fatality also remains inconclusive.

In Vietnam, EV-A71 was first detected in 2003. The first outbreak of HFMD was reported in 2005, with CV-A16 and EV-A71 being the leading causes, although EV-A71 was responsible for cases with neurological complications (Tu et al., 2007a). Since then, increasing numbers of severe cases of HFMD have occurred. In 2008, HFMD became a notifiable disease, and on average 10 000 cases were recorded annually between 2008 and 2010. In 2011–2012, there was a dramatic increase in hospitalized and fatal cases, with a total of over 200 000 children admitted and more than 200 deaths (Khanh et al., 2012). HFMD currently occurs throughout the country, but the highest burden of disease is recorded in the southern provinces in terms of absolute numbers and proportions of severe and fatal cases (Khanh et al., 2012).

A better understanding of the underlying aetiology of HFMD is needed for optimal therapeutic selection and vaccine development, but data on the current situation in Vietnam are limited. Most previously published work has focused on hospitalized patients with EV-A71-associated disease, and the epidemiology and aetiology of milder disease is poorly characterized. However, in recent years, clinical and national surveillance data from the region have shown the emergence of other EV-A serotypes (including CV-A6 and CV-A10) as the cause of hospitalized cases of HFMD, including cases of severe disease (Xing et al., 2014, Yang et al., 2014).

Until now, most studies have focused on severe disease and inpatients. In the present study >1500 patients were enrolled, covering a wider spectrum of severity and including outpatients. This article presents a detailed characterization of HFMD in southern Vietnam. The aim was to describe the aetiological agents associated with HFMD occurring between July 2013 and July 2015, to compare the associated epidemiological and clinical characteristics of HFMD in patients infected by different pathogens, and to identify potential risk factors associated with progression to more severe illness.

## Materials and methods

### Study setting

A prospective observational study was conducted in the outpatient clinics, clinical wards, and paediatric intensive care units (PICU) of three tertiary referral hospitals in southern Vietnam: Children’s Hospital 1, Children’s Hospital 2, and the Hospital for Tropical Diseases, all located in Ho Chi Minh City. For outpatient enrolment, a dedicated study room was assigned at the outpatient clinic of each hospital and patients were enrolled daily between 7.00 am and 4.00 pm. Clinically diagnosed HFMD patients seen by clinicians in other rooms were sent to the study room for screening. For inpatient enrolment, new patients were invited to participate in the study by study doctors on the clinical wards and in the PICU.

### Patient enrolment

All patients presenting to the three study hospitals between July 2013 and July 2015 with a clinical diagnosis of HFMD according to the guidelines of the Vietnamese Ministry of Health (Vietnam Ministry of Health, 2012) were eligible to participate in the study.

### HFMD case classification

Patients were classified as having a severe or non-severe illness using a grading system issued by the Vietnamese Ministry of Health based on Taiwanese guidelines using clinical signs and symptoms to guide hospital management (Huang et al., 1999). Briefly grade 1 includes patients with mouth ulcers or vesicles/papules on the hands, feet or buttocks, with or without mild fever (<39 °C). Grade 2 includes features of central nervous system (CNS) involvement, such as myoclonus, fever >39 °C, or ataxia. Grade 2 is divided into subgroups according to the reporting of myoclonus: 2A is myoclonus reported by the parents/care-givers only and grade 2B is when myoclonus is observed by a physician. Grade 2B is divided into two further subgroups: 2B1 is considered when myoclonus is observed by medical staff or there is a history of myoclonus and lethargy or pulse higher than 130 bpm; 2B2 is considered when ataxia, nystagmus, limb weakness, cranial nerve palsies, persistent high fever, or pulse higher than 150 bpm is recorded. Grade 3 includes autonomic dysfunction with sweating, hypertension, tachycardia, and tachypnea. In grade 4 disease, additional cardiopulmonary compromise with pulmonary oedema or shock syndrome are observed (Khanh et al., 2012). Patients with grade 2A and above need to be admitted to the hospital and patients with grade 2B and above are monitored in the ICU or emergency room of the infectious diseases ward (Supplementary material, Appendix 1). In this study severe HFMD was defined as grade 2B, 3, and 4.

### Clinical data and samples

Demographic and clinical data were recorded for all patients on enrolment to the study and daily until discharge or day 7 for inpatients (whichever was sooner), using standard paper case record forms (CRFs). For outpatients, similar data were collected by telephone on days 2, 4, and 6. Inpatients who were discharged before day 7 and all outpatients returned for a follow-up visit between days 7 and 14 after enrolment. CRF completion was double-checked before entering data into an electronic data entry system.

Rectal and throat swabs were collected at enrolment, in addition to the baseline haematology and biochemistry data obtained as part of routine care. An additional blood sample was collected at the follow-up visit. The enrolment process is described in detail in the Supplementary material (Appendix 2A).

### Enterovirus detection and serotype determination

Collected swabs were tested with a multiplex real-time RT-PCR using previously published generic EV and EV-A71 primers and probes (Thanh et al., 2015). For swab samples, rectal swabs were tested if throat swabs were negative (Thanh et al., 2015). Samples that were EV-positive but EV-A71-negative were then subjected to a genotyping step using a previously described VP1 sequencing assay (Perera et al., 2010, Kroneman et al., 2011), targeting the most variable and antigenic region of the viral genome. The VP1 sequences obtained were first assembled and then manually edited using Contig Express, a component of Vector NTI Suite 7 (Informax Inc., NY, USA). Finally, the sequences were analysed using an online EV typing tool available at http://www.rivm.nl/mpf/enterovirus/typingtool/to determine specific enterovirus serotypes (Kroneman et al., 2011).

### Statistical analysis

Data were analysed with R 3.2.5 (R Development Core Team, 2010). Missing data were tested for ‘missing at random’. As no variable was ‘missing not at random’ and the missing data rate was less than 20%, cases with missing data were removed (list-wise deletion). Continuous variables were expressed using the median and interquartile range (IQR) and categorical variables as the frequency. Fisher’s exact test with *p*-value by Monte-Carlo simulation, the *t*-test, the Kruskal–Wallis test, and the independent test were used to test categorical, continuous (normal distribution), continuous (skewed distribution), and categorical variables with ordinal variables, respectively. Multiple comparisons were adjusted using Bonferroni–Holm correction. To determine independent factors associated with disease progression, a primary analysis was first performed by comparing all variables of non-progressing and progressing patients. Patients who progressed to a more severe HFMD grade during hospitalization were divided into two groups for analysis: group 1 comprised those progressing to severe illness (i.e., from grade 1/2A to grade 2B1/2B2/3/4) and group 2 comprised those progressing to cardiopulmonary complications (i.e., from grade 1/2A/2B1/2B2 to grade 3/4). All factors with *p*-values <0.05 were tested in a generalized linear regression model. Variables were backward-selected and all variables with a *p*-value of >0.05 were removed.

## Results

### Patients and characteristics

Between July 2013 and July 2015, a total of 1547 patients were enrolled in the study. Baseline characteristics are given in Table 1. Patients were enrolled relatively early after the onset of symptoms (median 1 day, IQR 1–2 days). The median age of the children was 21.2 months (IQR 12.4–25.7 months). A total of 1423 (92%) patients had mild HFMD (grade 1 or 2A) on admission/enrolment and 1355 (87.6%) patients were classified as having mild disease at discharge, with 119 patients progressing to a higher grade during hospitalization (Table 1). Three patients were discharged with limb weakness; no fatalities were recorded in the study group (Supplementary material, Appendix 2B).

**Table 1:** Patient characteristics and comparison between inpatients and outpatients.^a^

	Total (*n* = 1547)	Outpatients (*n* = 590)	Inpatients (*n* = 957)	*p*-Value^b^
Demographics				
Sex (male)	933 (61.3)	344 (58.3)	589 (61.5)	0.2
Age (months)	21.2 (12.4–25.7)	20.2 (13.2–30.3)	16.8 (12.1–24)	***
Ho Chi Minh City origin	893 (57.7)	357 (60.5)	536 (56)	0.08
Illness day on admission (days)	1.4 (1–2)	1.2 (1–2)	1.5 (1–2)	***
Duration of hospital stay (days)	4.1 (2–5)	NA	4.1 (2–5)	NA
Clinical signs and symptoms				
Fever	1311 (73.2)	377 (63.9)	755 (78.9)	***
Rash	1310 (85.6)	541 (91.7)	769 (82.3)	***
Type of rash				***
Mostly vesicular	63 (4.9)	46 (8.5)	17 (2.2)	*
Mostly macular	601 (46.2)	215 (39.7)	386 (50.9)	
Both	636 (48.9)	281 (51.8)	355 (46.8)	
Mouth lesion	1345 (88.2)	506 (85.8)	839 (89.7)	*
Lips	64 (5)	61 (10.3)	3 (0.4)	***
Tongue	512 (39.9)	188 (31.9)	324 (46.8)	***
Palate	959 (74.6)	434 (73.6)	525 (75.8)	0.5
Buccal	256 (20)	201 (34.1)	55 (7.9)	***
Headache	22 (1.9)	19 (5.5)	3 (0.4)	***
Cough	347 (22.5)	207 (35.1)	140 (14.7)	***
Runny nose	326 (21.2)	214 (36.3)	112 (11.8)	***
Conjunctivitis	17 (1.1)	12 (2)	5 (0.5)	*
Vomiting	331 (21.4)	145 (24.6)	186 (19.5)	*
Diarrhoea	86 (5.6)	33 (5.6)	53 (5.6)	1
Drowsiness	35 (2.3)	9 (1.5)	26 (2.8)	0.1
Irritability	213 (13.8)	145 (24.6)	68 (7.2)	***
Myoclonus	261 (16.9)	64 (10.8)	197 (20.6)	***
Sweating	13 (0.84)	6 (1)	7 (0.7)	0.5
Lethargy	12 (0.8)	2 (0.3)	10 (1.1)	0.1
Ataxia	4 (0.4)	0	4 (0.4)	1
Tremor	30 (3.1)	0	30 (3.2)	1
Nystagmus	3 (0.3)	0	3 (0.3)	1
Limb weakness	5 (0.5)	0	5 (0.5)	1
Temperature (°C)	37.5 (35.6–38.2)	37 (36.9–37.5)	38 (37.4–38.5)	***
Pulse (bpm)	120 (110–130)	110 (100–110)	124 (120–130)	***
Systolic blood pressure (mmHg)	90 (85–90)	90 (80–90)	90 (90–97)	***
Diastolic blood pressure (mmHg)	55 (50–60)	50 (50–60)	60 (55–60)	***
Body mass index (kg/m^2^)	17.5 (15.6–18.6)	17.7 (16–18.7)	17.4 (15.5–18.5)	0.2
Blood results				
White blood cell count (10^9^/l)	12.5 (9.7–15.9)	11 (8.8–13.6)	13.6 (10.7–17.1)	***
Neutrophil (%)	48.7 (38.4–59.3)	46.2 (37.1–57.5)	50.3 (39.5–60.6)	***
Lymphocyte (%)	37.6 (27.8–47.7)	39.2 (29.3–48.2)	36.5 (26.9–46.5)	**
Platelet count (10^9^/ml)	300 (248–359)	306 (256.5–354)	296 (241–364)	0.4
Blood glucose (mg/dl)	99 (83–114)	112 (102–126)	88 (76–101)	***
C-reactive protein (mg/l)	7.6 (2.4–22)	3 (1–7.5)	13 (6–28.1)	***

NA, not applicable.

a Note: data are presented as the number (%) for categorical variables and as the median (interquartile range) for continuous variables; denominators may vary slightly.

b *p*-Values: *p < 0.05, **p < 0.01, ***p < 0.001.

### Enterovirus serotype detection and monthly distribution

**Figure 1:**
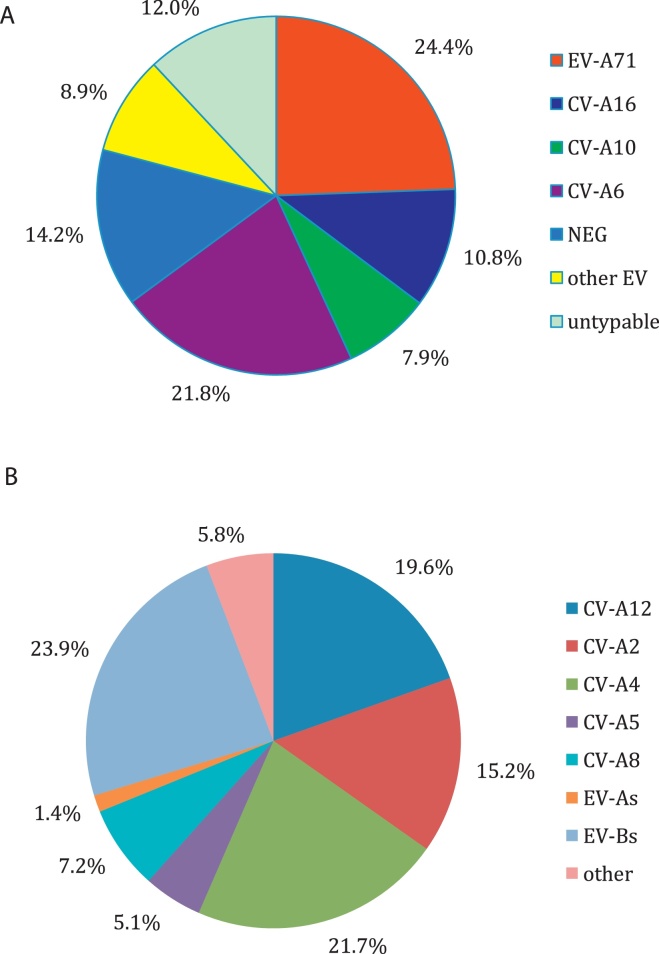
A) pie chart showing the proportions of four dominant genotypes of EV-A detected among 1547 patients with clinical HFMD enrolled between July 2013 and July 2015; B) pie chart showing the proportions of other genotypes of EV detected among 138 patients with clinical HFMD enrolled between July 2013 and July 2015.

Overall, 1327 patients (85.8%) were positive for enterovirus by multiplex real-time PCR. Serotyping and specific PCR analyses identified a total of 20 EV serotypes in HFMD patients in southern Vietnam during the study period (Figure 1A, B). The four dominant serotypes were EV-A71 (*n* = 378, 24.4%), CV-A6 (*n* = 337, 21.8%), CV-A10 (*n* = 122, 7.9%), and CV-A16 (*n* = 167, 10.8%), with EV-A71 being the most common (Figure 1A). The predominant sub-genogroup of EV-A71 in this cohort was B5 (unpublished data).

Other EV serotypes were detected in 138 patients, with CV-A4 being identified in 21.7%, followed by CV-A12 in 19.6%, CV-A2 in 15.2%, CV-A8 in 7.2%, CV-A5 in 5.1%, other enterovirus A in 1.4%, and enterovirus B in 23.9% (Figure 1B).

**Figure 2:**
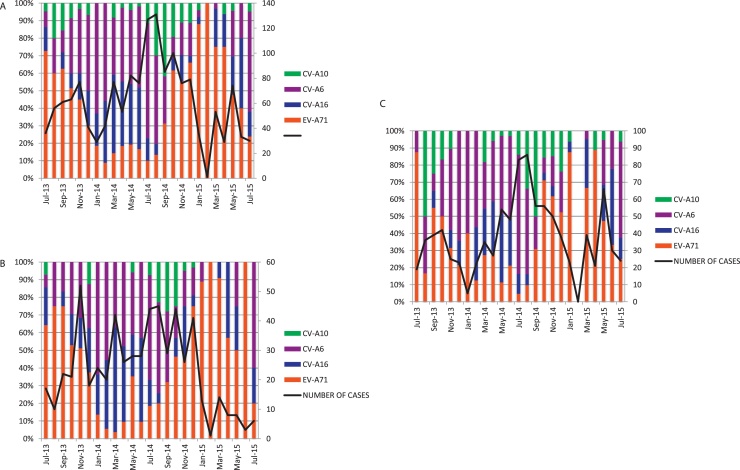
Monthly distribution of enterovirus genotypes over the study period (July 2013 and July15); A) all 1547 out- and inpatients; B) 590 outpatients and C) 947 inpatients.

Temporally, the four most common enterovirus serotypes (EV-A71, CV-A6, CV-A10, and CV-A16) replaced each other over the entire study period (Figure 2A). This phenomenon was seen in both the inpatient and outpatient groups (Figure 2B, C).

### Demographics, clinical characteristics, and laboratory data by pathogens

When stratifying the data by pathogens, there was considerable homogeneity in terms of demographics, clinical characteristics, and laboratory data between patients infected with the different enterovirus A serotypes. Variables for which there were statistically significant differences between patient groups are shown in Table 2.

**Table 2:** Clinical characteristics and laboratory results by pathogen.^a^

Characteristic	CV-A10 (*n* = 122)	CV-A16 (*n* = 167)	CV-A6 (*n* = 337)	EV-A71 (*n* = 378)	*p*-Value
Age (months)	14.3 (10.8–19.7)^b^	19.1 (14.2–27)	16.5 (11.9–22.4)	21.9 (14.2–32.1)	<0.001
Length of hospital stay (days)	3 (2–5)	3 (2–4)	3 (2–5)	4 (3–6)^b^	<0.001
Myoclonus	15 (12%)	18 (11%)	49 (15%)	90 (24%)^b^	0.002
Irritability	13 (11%)	18 (11%)	49 (15%)	89 (24%)^b^	<0.001
Tremor	0 (0)	0 (0)	1 (1%)	25 (12%)^b^	<0.001
Erythema	78 (64%)^b^	155 (93%)	330 (98%)	345 (95%)	<0.001
Mouth ulcers	117 (96%)^b^	156 (93%)	294 (87%)	311 (85%)	<0.001
White blood cell count (10^9^/l)	14.65 (12.18–18.1)^b^	12.2 (9.2–14.5)	12.5 (9.9–16.6)	11.7 (9.5–14.7)	<0.001
Platelet count (10^9^/l)	294 (250–364)	298 (251–342)	308 (249.7–359.2)	309 (261–376.75)	0.026
Glucose (mg/dl)	94.95 (82–111)	99.5 (81.1–112.2)	101 (85.36–117.5)	102 (86–118)	0.01
C-reactive protein (mg/l)	17.35 (6–34.23)^b^	7 (1.4–20)	10 (4–26)	5 (1.2–10)	<0.001

CV, Coxsackievirus; EV, enterovirus.

a Note: data are presented as the number (%) for categorical variables and as the median (interquartile range) for continuous variables; denominators may vary slightly. *p*-Values are adjusted for multiple comparison.

b Factors that are significantly different when compared with other groups.

Patients with EV-A71-associated HFMD were significantly older than the others (21.9 months, IQR 14.2–32.1 months), while patients with CV-A10-associated HFMD were the youngest (14.3 months, IQR 10.8–19.7 months) (Table 2).

**Figure 3:**
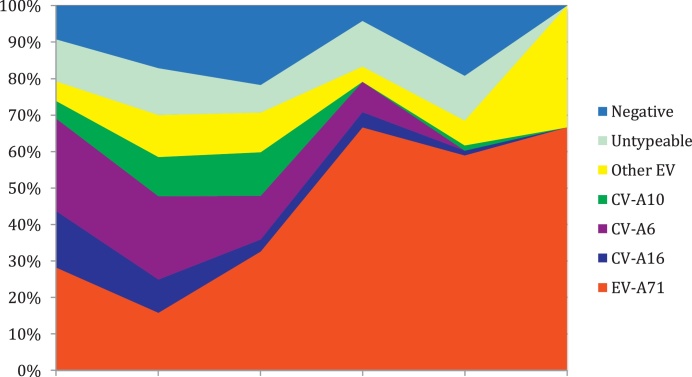
Distribution of pathogen detected by severity.

EV-A71 was detected at a higher frequency among patients with severe disease (grade 2B or more) compared to the other viruses (*p <* 0.01) (Figure 3). Likewise, other clinical presentations reflecting clinically severe HFMD (including duration of hospital stay, myoclonal jerks, tremors, and irritability) were more often observed among EV-A71-infected patients (Table 2). Compared with the other groups, CV-A10-infected patients had fewer skin lesions but more often had mouth ulcers (78/122 (64%) and 117/122 (96%), respectively (Table 2)). CV-A10-infected patients also had higher values for both white blood cell count and C-reactive protein (CRP) than the other groups (Table 2).

### Comparison between inpatients and outpatients

There were considerable differences in terms of demographics, clinical presentations, and laboratory test results between inpatients and outpatients (Table 1), while the aetiological patterns and monthly distribution of the detected enterovirus serotypes were similar in the two groups (Figure 2B, C). Notably, outpatients were older, more likely to have a rash, and had higher blood glucose levels than inpatients (Table 1). Meanwhile, inpatients presented to the hospital at a later time of illness, had a higher temperature, and had a higher white blood cell count and CRP than outpatients (Table 1).

### Factors associated with progression to severe disease

One hundred and nineteen inpatients (8.8%) progressed to a more severe grade of HFMD, of whom 98 (6.3%) progressed from non-severe grades to severe grades. A further 21 patients progressed within the mild grade category, i.e. from grade 1 to grade 2A (Table 3). Patients progressing from mild to severe grades were less likely to have a rash and mouth lesions on admission than those who did not. The types of rash also differed between the two groups. Vesicular and mixed rashes were seen more frequently in the non-progressing group, while progressing patients more often presented with a macular rash (Table 4). Myoclonus, tremor, and limb weakness at presentation were also less likely to be seen in the non-progressing group. Patients who progressed to the most severe grades (grade 3 and 4) with cardiopulmonary complications and/or autonomic dysfunction had a higher incidence of drowsiness and lethargy at presentation. Patients in this group also presented with a higher heart rate and systolic blood pressure, in addition to higher blood glucose and lower CRP than non-progressing patients or patients with milder disease (Table 4).

**Table 3:** Number of progressing cases.^a^

^a^Grey squares indicate patients with the same grade recorded at admission and discharge (non-progression).

^b^Patients who progressed from low grades to a higher grade (more severe) during their hospital stay.

**Table 4:** Comparison between progressing and non-progressing cases.^a^

	Did not progress from non-severe to severe (*n* = 859)	Progressed from non-severe to severe (*n* = 98)	*p*-Value^1^	Did not progress to cardiopulmonary complication (*n* = 907)	Progressed to autonomic dysfunction and/or cardiopulmonary complication (*n* = 50)	*p*-Value^2^
Demographics						
Sex (male)	517 (61.7)	72 (60.5)	0.8	558 (61.5)	31 (62)	1
Age (months)	16.6 (12.1–23.5)	18.7 (14.9–26.9)	0.2	16.7 (12.1–23.7)	18.7 (11.2–26.9)	0.7
Ho Chi Minh City origin	474 (55.2)	62 (63.3)	0.1	505 (55.7)	31 (62)	0.6
Illness day on admission (days)	1 (1–2)	2 (1–2)	0.002	1 (1–2)	2 (1–2)	0.05
Clinical signs and symptoms						
Rash	698 (83.4)	71 (73.2)	0.02	737 (83.3)	32 (65.3)	0.001
Type of rash			<0.001			<0.001
Mostly vesicular	13 (1.9)	4 (5.6)		15 (2.1)	2 (6.1)	
Mostly macular	339 (49.3)	47 (66.2)		360 (49.7)	26 (78.8)	
Both	335 (48.8)	20 (28.2)		350 (48.3)	5 (15.2)	
Mouth lesion	767 (91.5)	72 (74.2)	<0.001	808 (91.2)	31 (63.3)	<0.001
Lips	3 (0.5)	0	1	3 (0.4)	0	1
Tongue	305 (46.8)	19 (46.3)	1	314 (46.2)	10 (74.1)	0.06
Palate	499 (76.5)	26 (63.4)	0.06	518 (76.5)	7 (43.8)	0.005
Buccal	55 (8.4)	0	0.07	55 (8.1)	0	0.6
Headache	3 (0.4)	0	1	3 (0.4)	0	1
Cough	127 (14.9)	13 (13.5)	0.9	133 (14.8)	7 (13.5)	1
Runny nose	104 (12.2)	8 (8.2)	0.3	107 (11.9)	5 (10.2)	1
Conjunctivitis	4 (0.5)	1 (1)	0.4	4 (0.5)	1 (2)	0.2
Vomiting	167 (19.5)	19 (19.4)	1	175 (19.3)	11 (22)	0.6
Diarrhoea	50 (5.8)	3 (3.1)	0.3	51 (5.6)	2 (4.1)	1
Drowsiness	20 (2.4)	6 (6.6)	0.03	21 (2.4)	5 (11.6)	0.005
Irritability	61 (7.2)	7 (7.2)	1	66 (7.3)	2 (4.1)	0.6
Myoclonus	167 (19.5)	30 (30.6)	0.01	185 (20.4)	12 (24)	0.6
Sweating	6 (0.7)	1 (1)	0.5	7 (0.8)	0	1
Lethargy	7 (0.8)	3 (3.1)	0.08	7 (0.8)	3 (6)	0.01
Ataxia	4 (0.5)	0	1	4 (0.5)	0	1
Tremor	22 (2.6)	8 (8.8)	0.005	29 (3.2)	1 (2.3)	1
Nystagmus	3 (0.4)	0	1	3 (0.3)	0	1
Limb weakness	2 (0.2)	3 (3.3)	0.008	4 (0.5)	1 (2.4)	0.2
Temperature (°C)	38 (37.3–38.5)	38 (37.5–38.7)	0.4	38 (37.4–38.5)	37.8 (37.5–38.2)	0.3
Pulse (bpm)	122 (120–130)	130 (122–142)	<0.001	124 (120–130)	130 (120–142)	0.001
Systolic blood pressure (mmHg)	90 (90–95)	98 (90–104.5)	<0.001	90 (90–95)	98 (90–105)	<0.001
Diastolic blood pressure (mmHg)	60 (55–60)	60 (55–60)	0.5	60 (55–60)	60 (54–60)	0.3
Body mass index (kg/m^2^)	17 (15.5–18.6)	16 (15.5–18.2)	0.2	16.8 (15.5–18.6)	16.2 (14.9–17.9)	0.1
Blood results						
White blood cell count (10^9^/l)	13.6 (10.6–17.2)	13.1 (11.5–16.5)	1	13.6 (10.6–17.2)	13.2 (11.7–17.1)	0.7
Neutrophil (%)	50 (39.5–60.4)	45.6 (40.6–63.1)	0.054	50.2 (39.5–60.5)	54.5 (40.1–63.3)	0.3
Lymphocyte (%)	37 (27–46.6)	33 (25–45.8)	0.06	36.7 (27.1–46.4)	35.2 (23.9–48.2)	0.3
Platelet count (10^9^/ml)	294 (237.5–361.5)	320 (277–412)	0.051	295 (240–364)	322 (299.8–383.5)	0.1
Blood glucose (mg/dl)	87 (75–100)	97 (84.3–108.5)	<0.001	88 (75–101)	94 (85–106.7)	0.008
C-reactive protein (mg/l)	14 (6–29)	6 (2.1–21)	<0.001	13.2 (6–28.2)	6 (3–22.5)	0.004

a Note: data are presented as the number (%) for categorical variables and as the median (interquartile range) for continuous variables. *p*-Value^1^: *p*-values for cases that progressed from a non-severe grade to a severe grade. *p*-Value^2^: *p*-values for cases that progressed from a grade without autonomic dysfunction/cardiopulmonary involvement to a grade with autonomic dysfunction/cardiopulmonary involvement. The progressed from non-severe to severe group included patients admitted to the hospital with grade 1 or 2A who then progressed to grade 2B or higher. The progressed to autonomic dysfunction and/or cardiopulmonary complication group included patients admitted to the hospital with grade 1/2A who then progressed to grade 3 or grade 4.

Multivariate analysis showed that admission features of vesicular rash, increased blood pressure, high heart rate, and elevated blood glucose were associated with an increased risk of progression to severe grades (Table 5). For every 10 bpm increase in heart rate, the risk of progression increased by 0.54 (95% confidence interval 1.2–2). Similarly, for every 10 mmHg increase in systolic blood pressure, the risk of progression increased by 1.3 (95% confidence interval 1.4–3.8). However, when analysing only cases who progressed to very severe disease (grade 3 and 4, with autonomic dysfunction and/or cardiopulmonary complications), the presence of a skin rash was associated with protection from progression. When rash was present, a macular rash was significantly associated with an increased risk of progression (Table 6).

**Table 5:** Multivariate analysis of factors associated with disease progression from non-severe (grade 1–2A) to severe (all severe cases, grade 2B or higher).^a^

Factors	OR (95% CI)	*p*-Value
Type of rash (vesicular)	8.7 (1.4–43)	0.01
Blood glucose	2.9 (1.04–8.3)	0.04
Pulse	1.54 (1.2–2)	<0.001
Systolic blood pressure	2.3 (1.4–3.8)	0.001

OR, odds ratio; CI, confidence interval.

a Note: in the model, laboratory values of blood glucose were calculated by ln_2_. For pulse and systolic blood pressure, changes were evaluated for every 10 bpm and 10 mmHg, respectively.

**Table 6:** Multivariate analysis of factors associated with disease progression from non-severe (grade 1–2A) to severe with autonomic dysfunction and/or cardiopulmonary complications (grade 3–4).

Factors	OR (95% CI)	*p*-Value
Type of rash (macular)	3.2 (1.09–11.7)	0.04
Having skin lesions	0.03 (0.0007–1.15)	0.03

OR, odds ratio; CI, confidence interval.

## Discussion

This article reports a novel longitudinal study and in-depth analysis of the aetiology and associated clinical phenotypes of both inpatients and outpatients with HFMD admitted to three tertiary referral hospitals in southern Vietnam, a region where the majority of HFMD cases in Vietnam have been reported to date. Between July 2013 and July 2015, a total of 1547 HFMD patients were recruited. Between 2011 and 2012 Vietnam experienced an explosive outbreak of HFMD in terms of both hospitalized cases and fatal cases, with over 200 000 cases and 200 deaths (Khanh et al., 2012). The number and proportion of severe cases recorded in the present study was less than anticipated based on the previous 2 years of observations. Whilst the underlying mechanism remains unknown, previous research by the present authors showed that genetically EV-A71, the main cause of severe HFMD, had undergone a switch from sub-genogroup C4 to sub-genogroup B5 by the end of 2012 (Yi et al., 2017, Geoghegan et al., 2015), and since then B5 has continued to circulate in southern Vietnam. Intriguingly, the cyclical 2–3 year pattern of HFMD and its pathogens reported from Japan and Malaysia has not been seen in Vietnam (NikNadia et al., 2016, Iwai et al., 2009).

This study is one of the few to record the characteristics of mild cases of HFMD, finding a significant proportion of EV-A71 among these non-severe cases (168/590, 28.5%). The proportion of EV-A71-infected patients in mild and moderately severe cases (grade 1 and 2A) was remarkable in this study, with a detection rate of around 20%. All of these patients recovered completely without any complications. On the other hand, as EV-A71 was detected more frequently among severe patients, a higher rate of neurological symptoms among EV-A71-positive patients was also found.

This is the first study to report CV-A6-associated HFMD, previously not detected in Vietnam, including in the 2005 HFMD outbreak (Tu et al., 2007b); indeed this was the second most frequently detected pathogen after EV-A71. From October 2013 to October 2014, CV-A6 emerged, replacing CV-A16 and together with EV-A71 becoming the two most common pathogens in southern Vietnam, similar to reports from mainland China. Likewise other countries worldwide have also described the simultaneous emergence of CV-A6 (Yang et al., 2014, Lu et al., 2012, Kobayashi et al., 2013, Hayman et al., 2014, Hongyan et al., 2014, Ramirez-Fort et al., 2014).

The four main serotypes (EV-A71, CV-A6, CV-A16, and CV-A10) continuously varied in dominance and detection rates and replaced each other during the study period, while accounting for 1005/1327 (75.7%) of all EV-positive HFMD cases. This suggests complex patterns of aetiology and serotype replacement among HFMD patients, and emphasizes the need for active surveillance of circulating pathogens and to develop bivalent/multivalent vaccines to control HFMD. To support this, it is also important to assess the inter-relatedness among HFMD-causing pathogens as well as between other enteroviruses, particularly the level of cross-immunity in natural infection and post-vaccination.

Several differences in clinical features of the cases were detected. Inpatients presented significantly less often with rash than outpatients. This may represent either a health-seeking or diagnostic bias: either parents are prompted to go to hospital earlier when their children have a rash, or doctors only diagnose HFMD with subtle or no rash as HFMD when (characteristic) more severe symptoms of neurological involvement develop or on laboratory confirmation. Clinical phenotypes also appeared to differ according to the underlying viral aetiology. Patients testing positive for CV-A10 had a typical clinical presentation, with low frequency of skin rash but high frequency of mouth ulcers. Furthermore, they had higher white blood cell counts and CRP levels, which may suggest more severe illness or bacterial co-infection. As fever for more than 48 h is one of the admission criteria for HFMD according to the Vietnamese Ministry of Health guidelines, this finding suggest that patients with fever for more than 48 h only may not need to be admitted. Other signs of neurological involvement and/or laboratory test results such as CRP should also be considered for admission.

The lower median age of CV-A6- and CV-A10-infected patients suggests the absence of background (maternal) immunity and recent introductions into Vietnam, which merits further analysis into the evolutionary process of the pathogens and the level of cross-immunity between common HFMD-causing enteroviruses.

Similar to previous reports from Taiwan and China, several clinical features on admission that were indicative of progression to more severe stages of the disease were documented. A vesicular rash was shown to be associated with progression to more severe illness in the first regression model. However, due to the small number of events, the odds ratio confidence interval of this sign was relatively wide, making this finding inconclusive. The 50 patients who progressed to very severe disease (grade 3 and 4) less frequently presented with rash and mouth lesions and if presenting with rash, showed a macular rash as the predominant type. This could be explained by different types of rash caused by different pathogens. EV-A71, detected in 70% of very severe cases (grade 3 and 4), was more likely to be associated with a macular rash (72% of 378 EV-A71 cases in this study; data not shown), while other non-EV-A71 enteroviruses, dominant among patients with milder disease, were more often associated with a vesicular rash (67% of 1169 non-EV-A71 cases; data not shown).

The study approach, including inpatients and outpatients attending all three referral hospitals for HFMD treatment in the southern part of Vietnam, was unique and comprehensive and allowed the epidemiological patterns of HFMD to be captured. However, this study is subject to the following limitations. First, in terms of the clinical aspects, building and validating a prognostic model for severe HFMD was unachievable due to the unexpectedly low numbers of severe cases admitted during the study period and the small numbers of events (grade progression during admission), and only factors potentially associated with progression of severity could be identified, without validation. These factors will need to be validated in another HFMD cohort. Second, the case distribution could have been biased by the study setting and study staff capacity, especially for outpatients, as parents of mild cases may prefer to attend private clinics or lower level hospitals, and even if they had attended the study hospitals, the study staff may not have been able to approach all of them (i.e., patients came to the hospitals outside of working hours, when the study room was not operational). Therefore, the case distribution among enrolled cases may not reflect the distribution seen in the community. Third, although it was sought to minimize confounding factors by setting a quorum of patients enrolled every week, this quorum could not always be reached, since the number of patients who came to the three study hospitals varied with time. Therefore, the time-series findings should be interpreted with consideration. Lastly, the list-wise deletion method might have produced biased parameters, and other methods to impute missing data should be considered to minimize possible bias.

In conclusion, this study represents the most comprehensive descriptive HFMD study from Vietnam. The analysis of 1547 inpatients and outpatients revealed an overall dominance of EV-A71, especially among those with more a severe illness, but also among outpatients. Furthermore, it revealed detailed serotype emergence and replacement, including the emergence of CV-A6 and CV-A10 (in accordance with the global epidemiology), specific serotype-associated clinical features, and clinical and laboratory data associated with progression to more severe illness. These findings are essential for planning public health measures, particularly vaccine development and implementation. The clinical information could also be used as a reference for changing/modifying admission criteria to ensure that high-risk patients are closely monitored in the hospital and appropriate patients are hospitalized without placing a burden on the healthcare system through unnecessary admission. As it was not possible to build an effective model for progression, a further study with a larger sample size and more severe cases should be conducted to identify reliable risks factors for progression.
