# Supplementary material for: Clinical and aetiological study of hand, foot and mouth disease in southern Vietnam, 2013–2015: Inpatients and outpatients
Source: Int J Infect Dis. 2019 Mar;80:1–9. doi: 10.1016/j.ijid.2018.12.004 (PMC6403263; doi:10.1016/j.ijid.2018.12.004)
Supplement: Supplementary file 1 [file mmc1.docx]

| Classification | Signs/symptoms | Suggested Management |
| --- | --- | --- |
| Grade 1 | Oral ulcers and/or vesicular rash on the hands, feet, and/or the buttocks | OPD care, with advice sheet for family Careful observation for warning signs |
| Grade 2a | Grade 1 AND Myoclonic jerks observed by the family (not witnessed by medical staff) Legarthy, agitation/irritability Fever ≥ 39°C or ≥ 48 hours. Vomiting | Hospitalization Oral phenobarbitone Vital Signs Monitoring: every 4-6 hours following standard protocols |
| Grade 2b - Group 1 | Grade 1 AND Myoclonic jerks witnessed by medical staff or by the family (≥ 2 jerks/30 minutes or 1 jerk and stupor) Resting pulse rate > 130/min but <150/min (adjusted for fever*) | Admit to HDU/PCIU IV Phenobarbitone Antipyretics Vital Signs Monitoring: every 1-3 hours for minimum 6 hours until stable |
| Grade 2b - Group2 | Grade 1 AND witnessed myoclonic jerks accompanied by one of the following findings: Continuous limb tremor, limb weakness or paralysis, or drowsiness (provided no hypoglycemia) Resting pulse rate > 150 /min (adjusted for fever*) Fever ≥ 39.5 ⁰C (rectal) and unresponsive to antipyretics > 4 hours | Give oxygen IV Phenobarbitone Antipyretics Start IVIG – 2g/kg in two divided doses Check: FBC, CRP, blood sugar, and consider lumbar puncture Vital Signs Monitoring: every 1-3 hours for > 6hrs |
| Grade 3 | Serious complications in CNS or cardiopulmonary systems: Pulse >170 /min* Profuse sweating Stage 1 hypertension (SBP > 95th centile for age) Respiratory abnormalities: tachypnea, labored breathing Muscle hypertonia Coma (Glasgow coma score < 10) | Oxygenation Consider need for ventilation IV Phenobarbitone and IVIG Milrinone if stage 2 hypertension (SBP > 99th centile for age + 5 mm Hg) Dobutamine if HR> 170 bpm Consider additional fever control measures Invasive blood pressure monitoring Check: FBC, CRP, blood sugar, and consider lumbar Puncture Vital signs monitoring: every 30-60 mins for ≥ 6 hrs. |
| Grade 4 | Severe complications: Acute pulmonary edema Cardiac collapse | SpO2 < 92% with cannula oxygen 6 litres/min) Respiratory arrest or gasping respiration Intubation and ventilation IV Phenobarbitone Dobutamine; Fluid challenge Antipyretics, Access CVC plus invasive BP monitoring Vital signs monitoring: every 15-30 mins for ≥ 6 hrs. |

**Appendix 1: Vietnam Ministry of Health Guideline for management of Hand, Foot and Mouth Disease.**
