# Supplementary material for: Clinical and aetiological study of hand, foot and mouth disease in southern Vietnam, 2013–2015: Inpatients and outpatients
Source: Int J Infect Dis. 2019 Mar;80:1–9. doi: 10.1016/j.ijid.2018.12.004 (PMC6403263; doi:10.1016/j.ijid.2018.12.004)
Supplement: Supplementary file 2 [file mmc2.docx]

1

2

3

4

5

6

7

8-14

STUDY DAY

**Inpatients:**

Daily follow-up until discharge or day 8 of the study (whichever comes sooner)

Day 8-14: collect 2ml blood sample in hospital (while hospitalized or during follow-up visit)

**Outpatients:**

Phone call by study staff on day 2,4,6, asking for signs/symptoms/progression/admission

If admitted, continue with inpatient CRF

Day 8-14: collect 2ml blood sample during follow-up visit

**SCREENING**

**Inclusion criteria:**

- ≤ 12 years old

- Clinical diagnosed with HFMD

- Willing to come back for follow-up visit

- Written informed consent form

**For outpatients only:** <72h of illness

**Data collection using paper CRF**

History Throat + Rectal swabs

Demographics CBC, CRP, blood glucose

Signs and symptoms 2ml EDTA blood sample

**Appendix 2A: Study process**

Screening 658 patients

590 patients enrolled

57 refused to participate

11 did not meet inclusion criteria

590 patients available for analysis.

6 patients needed admission at grade 2A

22 patients refused to come back at day 14 but still could be reached by telephone

OUTPATIENTS

Screening 1036 patients

65 refused to participate

971 patients enrolled

14 patients withdrawn and did not allow the study staff to use their collected data

957 patients continued the study until discharge:

No fatal cases

3 patients with limb weakness

954 patients recovered completely

957 patients available for analysis.

12 patients did not come back at day 14

INPATIENTS

**Appendix 2B: Flowchart of patient enrolment.**
